# Copeptin as a Prognostic Marker in Acute Chest Pain and Suspected Acute Coronary Syndrome

**DOI:** 10.1155/2018/6597387

**Published:** 2018-01-24

**Authors:** Beata Morawiec, Damian Kawecki, Brygida Przywara-Chowaniec, Mariusz Opara, Piotr Muzyk, Lam Ho, Lui Chun Tat, Artur Gabrysiak, Olivier Muller, Ewa Nowalany-Kozielska

**Affiliations:** ^1^2nd Department of Cardiology, School of Medicine with the Division of Dentistry in Zabrze, Medical University of Silesia, Katowice, M. Skłodowskiej-Curie 10, 41-800 Zabrze, Poland; ^2^Department of Cardiology, Tuen Mun Hospital, 23 Tsing Chung Koon Rd, Tuen Mun, Hong Kong; ^3^Department of Accident and Emergency, Tuen Mun Hospital, 23 Tsing Chung Koon Rd, Tuen Mun, Hong Kong; ^4^Department of Cardiology, Lausanne University Hospital, rue du Bugnon 46, 1011 Lausanne, Switzerland

## Abstract

**Background:**

In patients admitted with chest pain and suspected acute coronary syndrome (ACS), it is crucial to early identify those who are at higher risk of adverse events. The study aim was to assess the predictive value of copeptin in patients admitted to the emergency department with chest pain and nonconclusive ECG.

**Methods:**

Consecutive patients suspected for an ACS were enrolled prospectively. Copeptin and high-sensitive troponin T (hs-TnT) were measured at admission. Patients were followed up at six and 12 months for the occurrence of death and major adverse cardiac and cerebrovascular events (MACCE).

**Results:**

Among 154 patients, 11 patients died and 26 experienced MACCE. Mortality was higher in copeptin-positive than copeptin-negative patients with no difference in the rate of MACCE. Copeptin reached the AUC 0.86 (0.75–0.97) for prognosis of mortality at six and 0.77 (0.65–0.88) at 12 months. It was higher than for hs-TnT and their combination at both time points. Copeptin was a strong predictor of mortality in the Cox analysis (HR14.1 at six and HR4.3 at 12 months).

**Conclusions:**

Copeptin appears to be an independent predictor of long-term mortality in a selected population of patients suspected for an ACS. The study registration number is ISRCTN14112941.

## 1. Introduction

The optimal risk stratification in patients with acute coronary syndrome (ACS) without persistent ST segment elevation is a priority for clinicians. Patients after an ACS remain on higher risk of the development of heart failure, further ischemic events, and death. It is crucial to identify possibly early those subjects who present with chest pain and are at higher risk, in order to enable the introduction of adequate strategy in optimal time frames. The guidelines for diagnosis and treatment of non-ST segment elevation ACS focus attention on early stratification of short- and long-term risk [1]. However, cardiac troponin (cTn), despite being the gold standard marker for diagnosis of myocardial injury, remains suboptimal in terms of early risk assessment. Due to the character of the release profile, repetitive blood sampling for cTn is needed to achieve satisfactory prognostic accuracy [2]. The introduction of high-sensitive assays allowed earlier stratification of patients but did not justify single measurement or risk stratification based solely on these assays. While looking for complemental markers that would increase the accuracy of cTn, we face a large variety of biomarkers [3]. It is of utmost importance from a practical point of view to indicate those that could be used for diagnostic and prognostic purposes simultaneously as early as possible after symptom onset. Copeptin, the C-terminal part of the prohormone for vasopressin, is a marker of acute endogenous stress, with rapid release pattern [4]. Several previous studies showed its good diagnostic accuracy in patients with an ACS [5–7]. Its potential prognostic role was described in patients with the history of an acute myocardial infarction (AMI) who developed heart failure [6, 8]. The strategy of combined use of copeptin and cTn, besides serving as a diagnostic tool, could identify patients with chest pain at higher risk for adverse events and the development of heart failure (HF) very early after symptom onset, thus enabling early triage decision. Despite the combination of copeptin and high-sensitive Tn (hs-Tn) was extensively described in terms of diagnosis of an ACS, there is insufficient data regarding its prognostic accuracy, especially in patients without persistent ST segment elevation. We therefore aimed to investigate if copeptin provides information on the prediction of outcome in patients admitted to tertiary cardiac centers with symptoms of ACS and nonconclusive ECG.

## 2. Methods

The Copeptin for Acute Coronary Syndrome (COPACS) is a prospective, investigator-initiated, observational study with the aim to evaluate the role of copeptin in the diagnostic and prognostic process in patients with acute chest pain to enhance rapid evaluation in the emergency department.

Details on the design and the chart of the study were widely described previously [9]. In brief, consecutive patients presenting to the emergency department of the 2nd Department of Cardiology in Zabrze, Medical University of Silesia, Katowice, Poland, were screened in 24/7 manner. Patients with chest pain lasting 5 minutes or more during the last 6 hours and the absence of persistent ST segment elevation in admission ECG were prospectively enrolled to the study. Major conditions with proved influence on copeptin elevation were regarded as exclusion criteria (e.g., end-stage renal disease, sepsis, anaemia, and hyponatremia). According to the tertiary character of the enrolling center, patients admitted to the emergency department conform a highly preselected population, referred mainly via regional hospital. In the structure of our health care system, high rate of patients with chest pain and without persistent ST segment elevation on ECG undergoes initial stratification in regional hospitals. Therefore, patients are referred and admitted to tertiary cardiac centers after initial qualification to invasive coronary angiography with longer delays from chest pain onset and high suspicion of an ACS. As an effect, first, vast majority of patients present later than six hours after chest pain onset and, second, high rate of patients is finally diagnosed with acute coronary syndrome.

The study protocol conforms to the ethical guidelines of the 1975 Declaration of Helsinki as reflected in a priori approval by the Ethical Committee of Medical University of Silesia (decision number KNW/0022/KB1/187/11). All patients gave their written informed consent before inclusion to the study. The study registration number is ISRCTN14112941.

The design of the study, data gathering, and analysis were conducted according to the STARD guidelines for studies of diagnostic/prognostic accuracy. All authors contributed to the work by participating in the design, collecting and analyzing the data, and writing the paper, and all accepted the final draft of the manuscript.

After inclusion, each patient underwent initial clinical examination which included physical examination, 12-lead electrocardiogram (ECG), echocardiographic examination, and standard laboratory tests (blood count, sodium, potassium, creatinine, GFR, C-reactive protein [CRP], and N-terminal pro-B type natriuretic peptide [NT-proBNP]). Hs-TnT, creatine kinase myocardial bound (CK-MB), and copeptine were measured at admission (T0). Copeptin was double-blinded until final adjudication of the diagnosis. Hs-TnT and CK-MB were afterwards measured at six hours (T6) and repeated according to clinical indications.

Initial diagnosis was set by the emergency physician and was verified by a supervisor cardiologist based on available data and the ESC guidelines [1]. All patients were stratified according to the GRACE 1.0 risk score.

Further, all included patients underwent routine diagnostic and therapeutic procedures as indicated in the ESC guidelines for non-ST segment elevation ACS [1] and according to the study design [9]. Final diagnosis of non-ST elevation myocardial infarction (NSTEMI), unstable angina (UA), or other causes of chest pain was set based on independent opinions of two cardiologists, after analysis of all available data and tests gathered during the hospital stay. In case of incoherence of their diagnosis, a third cardiologist was asked for opinion.

Copeptin was measured once, at admission (T0) from the blood sample managed according to the instructions of the manufacturer of the test. The measurement was performed using the BRAHMS Copeptin KRYPTOR kit on BRAHMS KRYPTOR compact plus analyzer (BRAHMS GmbH, Hennigsdorf, Germany)—detection limit at 4.8–500 pmol/l, 20% coefficient of variation (CV) at 12 pmol/l, and the 97.5th percentile for healthy population at 17.4 pmol/l. According to the general rule for the optimal cutoff for a marker at the 99th percentile of healthy population, copeptin was regarded as positive when ≥17.4 pmol/l, following available information provided by the manufacturer on the most compliant value (97.5th percentile) to that recommended in the guidelines [1, 10]. For secondary analysis, the concentration of 10 pmol/l was used as a cutoff [10].

Troponin T was measured at admission (T0), after 6 hours from admission (T6), and at further time points according to the decision of treating physician. Troponin T was measured using an Elecsys Troponin T hs STAT kit on a cobas e 411 analyzer (Roche Diagnostics GmbH, Mannheim, Germany) with a high-sensitive electrochemiluminescence method (limit of detection 3–10,000 ng/l, 99th percentile for healthy population 14 ng/l [95% CI 12.7–24.9 ng/l], and 10% CV 3 ng/l). Hs-TnT was regarded as positive when ≥14 ng/l, according to the manufacturer's indications and the guidelines [1].

The observational data of all patients were analyzed after six months and one year. Six-month follow-up was conducted in a phone call with the patient, relatives, or primary care physician and included the information on Canadian Cardiovascular Society (CCS) class, New York Heart Association (NYHA) class, and the occurrence of endpoints. At one year, during a visit in the outpatient unit, following data were gathered: CCS and NYHA classes, the occurrence of endpoints, echocardiogram with the assessment of EF, and blood draw for NT-proBNP.

Primary endpoint was defined as death of cardiovascular origin. Secondary endpoints were major adverse cardiac and cerebrovascular events (MACCE) combined with death of cardiovascular origin, nonfatal AMI, UA, repeated cardiac revascularization, and stroke. Patients were also screened for the occurrence of major bleeding.

Maximal hs-TnT/CK-MB was the maximal concentration of the biomarker measured during the hospital stay. Smoker was regarded as past if one was free of smoking for at least one year before admission. Familial history of coronary artery disease was positive if AMI, stroke, or cardiac death occurred in at least one first-degree female relative at the age of <55 years or male relative at the age of <65 years. Coronary artery disease was diagnosed in coronary angiogram if the stenosis of coronary artery was >75% (or >50% for left main).

Data were checked for normality of distribution with the Shapiro-Wilk test. Continuous variables are presented as mean, standard deviation (SD), or median (interquartile range [IQR]) and were compared with the Student *t*-test or Mann–Whitney test and ANOVA or Kruskal-Wallis test, depending on their distribution. Categorical variables are presented as *n* (%) and were compared with chi-square test. The correlation between copeptin and other parameters was assessed with Spearman's method. To evaluate the prognostic accuracy, the receiver operating characteristic (ROC) curves with areas under the curve (AUC) were used and compared with the *z* test. Survival curves for copeptin and hs-TnT were performed with the Kaplan-Meier method. The influence of biomarkers and preselected baseline, clinical, and procedural parameters on the occurrence of endpoints was calculated in the Cox proportional hazard regression model. The taxonomic analysis (focus analysis) was used as a supplementary method to analyze prognostic accuracy in a multivariate manner and is described in online Supplementary Material 1. The *p* value of <0.05 was assumed significant throughout all analyses. All analyses were performed with Statistica software, version 10PL (StatSoft Inc., Tulsa, OK, USA); GraphPad Prism, version 6.00 (GraphPad, La Jolla, California, USA); and platform R, version 3.0.2 (R Foundation for Statistical Computing, Vienna, Austria).

## 3. Results

During the study period, a total of 1665 were screened. After exclusion of 424 patients presented with STEMI and 1241 patients due to other exclusion criteria (40 patients with anaemia, 31 with hyponatremia, 13 with laboratory errors, 995 patients with chest pain out of inclusion criteria, and 8 patients due to withdrawal of the informed consent), a total of 154 patients were enrolled to the study. Mean age of the population was 63, SD was 12 years, and 65% were male. After adjudication of the final diagnosis, 105 patients (68%) were diagnosed with NSTEMI, 30 patients (20%) were diagnosed with UA, and 19 patients (12%) presented other causes of chest pain. Mean GRACE risk score was 124 (104–147) for the population. Copeptin was positive in 48 patients (31%) with the median of 11.56 (5.67–21.61) pmol/l.

Patients positive and negative for copeptin had a similar clinical profile. Groups differed significantly with age; patients were older in the copeptin-positive group. Regarding the risk profile, patients positive for copeptin had higher hs-TnT concentrations measured at every time point; higher six-hour and maximal concentration of CK-MB; higher admission NT-proBNP, CRP, and leukocytosis and lower GFR; and higher GRACE risk score. Patients with positive copeptin result had similar procedural characteristics and received equal medical therapy as copeptin-negative patients with significant differences with regard to in-hospital catecholamine administration, more frequent in copeptin-positive patients. Results are summarized in Table 1. Similar results were observed for patients positive and negative for copeptin with the cutoff 10 pmol/l and are presented in Supplemental Table S1.

Positive correlation of copeptin was found with age (*r* = 0.36 [0.18–0.52]), maximal hs-TnT (*r* = 0.24 [0.05–0.42]), six-hour and maximal CK-MB (*r* = 0.34 [0.16–0.5] and *r* = 0.35 [0.17–0.51], resp.), leukocytosis (*r* = 0.36 [0.18–0.52]), and the GRACE risk score (*r* = 0.48 [0.32–0.62]). Negative correlation reached statistical significance with GFR (*r* = −0.27 [−0.44; −0.08]) and EF (*r* = −0.3 [−0.46; −0.11]).

Follow-up was completed in 98% of patients at six months and 95% of patients at one year. Overall, 11 patients died (8%) and 26 (18%) experienced MACCE (11 deaths, 3 AMI, 10 PCI, and 2 strokes). No major bleeding was reported during the follow-up. Patients who died had significantly higher concentrations of copeptin than survivors (103 [21–168] pmol/l versus 11 [5.3–20] pmol/l, resp., *p* = 0.001), while there was no statistically significant difference in copeptin level between patients who experienced MACCE and other patients (17 [6.0–52] pmol/l versus 11 [5.4–20] pmol/l, resp., *p* = 0.19).

At one year, copeptin-positive patients had higher NT-proBNP concentrations than copeptin-negative patients (216 [140–457] pg/ml versus 147 [80–359] pg/ml, *p* = 0.049). They also had higher NYHA class with more patients in class III (20% versus 8%) and less in class I (50% versus 68%) than copeptin-negative patients (*p* = 0.014). Concentrations of NT-proBNP remained higher in copeptin-positive than in copeptin-negative patients when the cutoff 10 pmol/l was used (250 [118–567] pg/ml versus 135 [77–227] pg/ml, *p* = 0.003), and no statistically significant difference was observed in NYHA class between both groups (*p* = 0.10).

**Figure 1:**
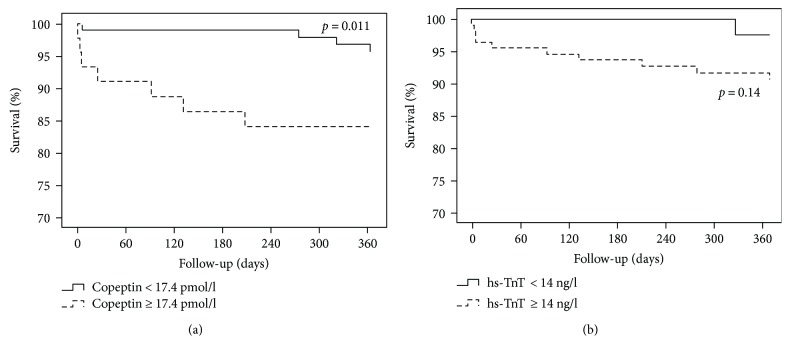
Survival curves or copeptin (a) and hs-TnT (b). Hs-TnT: high-sensitive troponin T.

According to Kaplan-Meier analysis, patients with negative copeptin levels had significantly better survival than copeptin-positive patients at six months (103/104 versus 41/47 patients, *p* = 0.001) and one year (98/102 versus 38/45 patients, *p* = 0.011) (Figure 1(a)). After dichotomization with the cutoff 10 pmol/l, similar results were observed at six months (69/71 versus 67/76, *p* = 0.033) and one year (71/71 versus 73/80 patients, *p* = 0.011) (Supplemental Figure S1). No statistically significant difference in survival was observed regarding hs-TnT levels throughout the follow-up (*p* = 0.10 and *p* = 0.14 at six months and one year, resp.) (Figure 1(b)).

Copeptin achieved a good prognostic accuracy in the ROC analysis at six months (AUC 0.86 [0.75–0.97], *p* < 0.001) and one year (AUC 0.77 [0.65–0.88], *p* = 0.003). It was higher when compared to hs-TnT and their combination at six months (0.62 [0.53–0.72], *p* = 0.002, versus 0.76 [0.53–0.99], *p* = 0.42, resp.) and one year (0.63 [0.52–0.75], *p* = 0.12, versus 0.63 [0.43–0.83], *p* = 0.24, resp.). Of note, the ROC analysis of hs-TnT did not reach statistical significance for the prediction of death at any time point. Additional analysis with the GRACE risk score for six-month prediction of death revealed that copeptin was not inferior to the GRACE risk score alone (0.86 [0.75–0.97] versus 0.96 [0.92–1.0], *p* = 0.09), and the highest AUC across all analyzed factors for six-month mortality was observed for the combination of copeptin with the GRACE risk score (0.98 [0.95–1.0], *p* < 0.001). It outperformed both of the predictors alone (*p* = 0.04) and hs-TnT (*p* < 0.001).

In the Cox regression analysis, patients with copeptin level ≥ 17.4 pmol/l were at significantly higher risk of death at six months (hazard ratio (HR) 14 (1.7–117), *p* = 0.014) and at one year (HR 4.32 [1.27–14.77], *p* = 0.02). Contrary, copeptin level ≥ 10 pmol/l was not a significant risk factor for death at six months (HR 61 [0.1–26.86], *p* = 0.19) nor at one year (HR 4.56 [0.99–21.11], *p* = 0.052). Hs-TnT measured at admission was not a predictor of mortality in the Cox analysis, neither was maximal concentration of hs-TnT. Statistical significance for the prediction of mortality was reached by age (HR 1.1 [1.04–1.18], *p* = 0.002) and GFR (HR 0.92 [0.89–0.95], *p* < 0.001). Detailed results are presented in Table 2. The combination of higher copeptin/older age/lower GFR identified a group with higher admission and follow-up NT-proBNP levels, lower baseline ejection fraction, and higher risk according to the GRACE risk score (Supplemental Table S2).

## 4. Discussion

This prospective, observational study assessed the prognostic role of copeptin and its combination with hs-TnT in consecutive patients with acute chest pain admitted to the emergency department of a tertiary cardiologic center.

The first finding is the high prognostic accuracy of copeptin in the prediction of mortality. According to previously published data [11, 12], we report higher mortality in patients with higher plasma levels of copeptin. Secondly, copeptin had significantly better prognostic accuracy than hs-TnT in studied population. There was no benefit from a combined use of copeptin and hs-TnT over copeptin alone. Thirdly, copeptin should be considered together with other, but still simple risk factors, especially age and renal function, while assessing prognosis. Lastly, we provide indirect evidence for the significant prognostic value of copeptin in the prediction of HF at long-term assessed with NT-proBNP, a recently strongly recommended diagnostic parameter of HF [13].

The prognostic value of copeptin in patients with chest pain is the field of research interest and constant growth. In this study, we add evidence on prognostic utility of copeptin as an early marker of adverse outcome in a specific population of highly preselected patients with high rate of finally diagnosed ACS, characteristic for tertiary centers. The decision on optimal type and time frame of the management is essential for non-ST segment elevation ACS and appropriate selection of patients in whom invasive interventions are likely to be beneficial. According to the guidelines [1], the triage of patients in such circumstances should be extensive; involve clinical status of patients, medical history, the dynamics of ECG, and biomarker concentrations; and be supported by different risk scoring systems, for example, the GRACE risk score. Despite the GRACE risk score is a validated tool in risk stratification [14], the practical utility is compromised in everyday clinical practice [15]. We provide evidence that supports very early risk assessment with single measure from a blood draw at admission. The results of the Cox analysis identified copeptin, age, and renal dysfunction as risk markers of poor prognosis at one year. Whether this combination of risk factors would play a wide practical role remains to be determined in a larger cohort.

The single measurement of copeptin at admission showed also a tendency to increase the prognostic accuracy of the GRACE risk score for death at six months. It confirms previous reports on good prognostic performance of copeptin in the prediction of death, however assessed in different clinical setting including STEMI [16] or derived from measurements at later time points [6]. Nevertheless, these results might serve as a background for further research on a simple and concise prognostic evaluation methodology, which would be of clinical benefit at the bedside, without the need for extensive, repetitive examinations or online calculations.

The combination of copeptin and hs-TnT merits consideration. The estimation of prognosis based on combined use of copeptin and hs-TnT was not clinically relevant. Surprisingly, hs-TnT alone was also not a significant predictor of long-term death. It is known that cTn directly correlates with infarct size [17] and the predictive value of cTn is higher for maximal concentrations measured during the hospital stay [18]. Of note is a correlation found for copeptin with maximal concentrations of hs-TnT in our study. This leads to speculate, first, that this is the reason why hs-TnT measured at admission had low prognostic value and, second, that copeptin might be considered an early indirect predictor of infarct size. The latter hypothesis was recently evaluated and confirmed in a population of STEMI patients [19]. As a consequence might be regarded the observation of predictive value of copeptin for the development of heart failure, with higher and diagnostic for HF levels of NT-proBNP among patients stratified with copeptin, age, and GFR as high risk. It was not reflected in the value of EF but it is known that levels of NT-proBNP might be elevated also in patients with preserved EF [20]. We would like, however, to prevent the reader from interpreting our results as a prove of direct relationship between copeptin and infarct size and/or heart failure and consider them as a hypothesis that warrants further research.

In conclusion, copeptin appears to be an independent predictor of long-term mortality in a selected population of patients suspected for an ACS. In addition, copeptin may be considered as an early marker for the identification of patients at higher risk of the development of HF at long term in this population. The outcomes warrant a confirmation in a larger cohort.

### 4.1. Limitations

The following limitations should be mentioned. The observational character of the study limits the interpretation of clinical benefit from risk assessment with copeptin. Further, studied troponin was the hs-TnT (Roche). It remains unknown if the use of other assays or other troponin would influence the outcomes. Next, in the study, we used a prespecified cutoff for copeptin at the 97.5th percentile, as the closest to that recommended by the guidelines (99th percentile) that was available from the manufacturer's resources; therefore, the outcomes are limited with regard to previously proposed values [6, 11]. Further studies are warranted to directly compare different cutoffs to identify the optimum for prognostic purposes.

## Figures and Tables

**Table 1:** Baseline characteristics in patients positive and negative for copeptin.

	Overall cohort (*n* = 154)	Copeptin < 17.4 pmol/l (*n* = 106)	Copeptin ≥ 17.4 pmol/l (*n* = 48)	*p* value
Baseline parameters and medical history				
Age (years)	63 (57–73)	62 (56–69)	65 (57–78)	0.04
Male sex	100, 65%	72, 65.5%	28, 58.3%	0.25
BMI (kg/m^2^)	28.7 (42.9–32.3)	28 (25–32)	29 (25–32)	0.93
CAD	67, 44%	50, 45.5%	17, 35.4%	0.17
Hypertension	114, 74%	75, 68.2%	39, 81.3%	0.17
Diabetes mellitus	42, 27%	28, 25.5%	14, 29.2%	0.72
PAD	4, 2.6%	3, 2.7%	1, 2.1%	0.79
Familial history of CAD	21, 14%	18, 16.4%	3, 6.3%	0.07
Current smoker	51, 33%	38, 34.5%	13, 27.1%	0.28
Past smoker	31, 20%	23, 20.9%	8, 16.7%	0.47
Dyslipidemia	67,44%	49, 44.5%	18, 37.5%	0.31
History of AMI	46, 30%	36, 32.7%	10, 20.8%	0.10
History of PCI	48, 31%	36, 32.7%	12, 25.0%	0.27
History of CABG	7, 4.5%	7, 6.4%	0, 0%	0.07
History of stroke	4, 2.6%	3, 2.7%	1, 2.1%	0.79
Baseline clinical status				
Heart rate (beats/min)	75 (66–88)	70 (65–80)	75 (70–85)	0.07
Systolic BP	140 (123–160)	140 (125–160)	140 (120–160)	0.54
EF (%)	55 (45–60)	55 (45–60)	55 (43–60)	0.56
NYHA class III or IV	4, 2.6%	1, 0.9%	3, 6.3%	0.06
Killip class				0.50
1	139, 90%	98, 89.1%	41, 85.4%	
2	14, 9.1%	7, 6.4%	7, 14.6%	
3	1, 0.6%	1, 0.9%	0, 0%	
4	0, 0%	0, 0%	0, 0%	
GRACE	124 (104–146)	120 (101–141)	131 (111–167)	0.03
Laboratory parameters				
Hs-TnT T0 (ng/l)	33 (13–143)	25 (11–125)	68 (30–177)	0.01
Hs-TnT T6 (ng/l)	75 (16–397)	32 (13–247)	234 (43–2284)	<0.001
Hs-TnT max (ng/l)	105 (23–530)	52 (17–274)	236 (74–2070)	<0.001
CK-MB T0 (IU/l)	20 (15–30)	19 (14–28)	22 (17–36)	0.06
CK-MB T6 (IU/l)	21 (14–45)	18 (13–32)	33 (18–107)	<0.001
CK-MB max (IU/l)	27 (18–53)	25 (17–42)	42 (25–111)	<0.001
NT-proBNP (pg/ml)	350 (163–1074)	289 (150–872)	491 (223–1979)	0.02
CRP (mg/l)	2.9 (1.3–5.5)	3 (1–5)	4 (2–7)	0.03
Leukocytosis (10^3^/*μ*l)	8.4 (6.9–10.3)	8 (6–9)	10 (8–12)	<0.001
Hemoglobin (g/dl)	14 (13–15)	14 (13–15)	14 (13–15)	0.34
GFR (ml/min/1.73m^2^)	92 (76–110)	93 (77–115)	90 (67–99)	0.08
In-hospital parameters				
Diagnosis of CAD	116, 75%	83, 75.5%	33, 68.8%	0.11
Medical therapy	38, 25%	25, 22.7%	13, 27.1%	0.64
PCI	90, 58%	60, 54.5%	30, 62.5%	0.49
CABG	33, 21%	25, 22.7%	8, 16.7%	0.33
Catecholamines	4, 2.6%	0, 0%	4, 8.3%	0.003
ASA	141, 92%	97, 88.2%	44, 91.7%	0.79
DAPT	99, 64%	68, 61.8%	31, 64.6%	0.96
*β*-Blocker	134, 87%	93, 84.5%	41, 85.4%	0.81
ACE inhibitor	126, 82%	91, 82.7%	35, 72.9%	0.07
Statin	135, 88%	95, 86.4%	40, 83.3%	0.26
Diuretic	45, 29%	31, 28.2%	14, 29.2%	0.97
Ca-blocker	39, 25%	27, 24.5%	12, 25.0%	0.98
Nitroglycerin	17, 11%	12, 10.9%	5, 10.4%	0.89
Final diagnosis				
Unstable angina	30, 20%	27, 24.5%	3, 6.3%	0.005
NSTEMI	105, 68%	65, 59.1%	40, 83.3%	0.007

Data presented as *n*, %, or median (25th–75th interquartile range). ACE: angiotensin-converting enzyme; AMI: acute myocardial infarction; ASA: acetylsalicylic acid; BMI: body mass index; BP: blood pressure; CABG: coronary artery bypass grafting; CAD: coronary artery disease; CK-MB: creatine kinase myocardial bound; CRP: C-reactive protein; DAPT: dual antiplatelet treatment; EF: ejection fraction; GFR: glomerular filtration ratio; GRACE: Global Registry for Acute Coronary Events; Hs-TnT: high-sensitive troponin T; NSTEMI: non-ST segment elevation myocardial infarction; NT-proBNP: N-terminal pro-B-type natriuretic peptide; NYHA: New York Heart Association; PAD: peripheral artery disease; PCI: percutaneous coronary intervention.

**Table 2:** The Cox regression model for death at 6 and 12 months.

Characteristic	At six months			At one year		
	HR	95% CI	*p* value	HR	95% CI	*p* value
Copeptin ≥ 17.4 pmol/l	14.1	1.7–116.8	**0.01**	4.3	1.3–14.8	**0.02**
Age	1.3	1.1–1.5	**0.001**	1.1	1.04–1.2	**0.002**
Male sex	0.2	0.04–1.1	0.06	0.6	0.2–1.9	0.359
Diagnosis of NSTEMI	3.0	0.4–24.7	0.31	2.3	0.5–10.8	0.281
Prior AMI	1.0	0.2–5.0	0.96	1.4	0.4–4.8	0.591
Diabetes mellitus	1.1	0.2–5.8	0.89	1.1	0.3-4.1	0.909
GFR	0.9	0.86–0.95	<**0.001**	0.99	0.9–0.95	<**0.001**
EF	1.0	0.9–1.0	0.09	0.95	0.9–0.99	**0.026**
Admission NYHA class 3 or 4	7.7	1.3–46.1	**0.03**	11.8	1.9–72	**0.008**
Hs-TnT ≥ 14 ng/l at admission	33.6	0.03–366,658	0.33	4.1	0.5–32.3	0.176
Hs-TnT mx	1.0	0.99–1.0	0.82	1.0	0.99–1.0	0.642

AMI: acute myocardial infarction; CI: confidence interval; EF: ejection fraction; GFR: glomerular filtration ratio; GRACE: Global Registry of Acute Coronary Events; HR: hazard ratio; hs-TnT mx: maximal concentration of high-sensitive troponin T; NSTEMI: non-ST segment elevation myocardial infarction; NYHA: New York Heart Association.

## Supplementary Materials

Supplementary Materials Table S1: baseline characteristics in patients positive and negative for copeptin with the cutoff 10 pmol/l. Table S2: outcomes of the taxonomic analysis. Figure S1: survival curves or copeptin with the cutoff 10 pmol/l. Figure S2: dendrogram—taxonomic analysis.
