# Supplementary material for: Copeptin as a Prognostic Marker in Acute Chest Pain and Suspected Acute Coronary Syndrome
Source: Dis Markers. 2018 Jan 24;2018:6597387. doi: 10.1155/2018/6597387 (PMC5829365; doi:10.1155/2018/6597387)
Supplement: Supplementary Materials — Table S1: baseline characteristics in patients positive and negative for copeptin with the cutoff 10 pmol/l. Table S2: outcomes of the taxonomic analysis. Figure S1: survival curves or copeptin with the cutoff 10 pmol/l. Figure S2: dendrogram—taxonomic analysis. [file 6597387.f1.pdf]

Copeptin as a prognostic marker in acute chest pain and suspected acute coronary syndrome

Online supplemental appendix

Beata Morawiec, MD, PhD[a]*, Damian Kawecki, MD, PhD[a]*, Brygida Przywara-Chowaniec, MD, PhD[a], Mariusz Opara, MD[a], Piotr Muzyk, MD[a], Lam Ho, MD, PhD[b], Lui Chun Tat, MD, PhD[c], Artur Gabrysiak[a], Olivier Muller, MD, PhD[d], Ewa Nowalany-Kozielska, MD, PhD, Prof[a]

both authors contributed equally to this publication and should be considered first author

[a] 2nd Department of Cardiology, School of Medicine with the Division of Dentistry, Zabrze, Medical University of Silesia, Katowice, M.-Skłodowskiej 10, 41-80 Zabrze, Poland

[b] Department of Cardiology, Tuen Mun Hospital, 23 Tsing Chung Koon Rd, Tuen Mun, Hongkong

[c] Department of Accident and Emergency, Tuen Mun Hospital, 23 Tsing Chung Koon Rd, Tuen Mun, Hongkong

[d] Department of Cardiology, University Hospital, Lausanne, Rue du Bugnon 46, 1011 Lausanne, Switzerland

Supplementary results for copeptin with the cut-off 10 pmol/l.

Table S1. Baseline characteristics in patients positive and negative for copeptin with the cutoff 10pmo/l.

	**Overall cohort n=154**	**Copeptin negative n=72**	**Copeptin positive n=82**	**p value**
**BASELINE PARAMETERS AND MEDICAL HISTORY**				
Age [years]	63 (57-73)	60 (54-67)	65 (59-75)	0.001
Male sex	100, 65%	46, 60.5%	54, 65.9%	0.80
BMI [kg/m^2]	28.7 (42.9-32.3)	28 (25-31)	29 (25-33)	0.15
CAD	67, 44%	36, 47.4%	31, 37.8%	0.13
Hypertension	114, 74%	46, 60.5%	68, 82.9%	0.007
Diabetes Mellitus	42, 27%	17, 22.4%	25, 30.5%	0.34
PAD	4, 2.6%	2, 2.6%	2, 2.4%	0.90
Familial history of CAD	21, 14%	13, 17.1%	8, 9.8%	0.14
Current smoker	51, 33%	23, 30.3%	28, 34.1%	0.77
Past smoker	31, 20%	17, 22.4%	14, 17.1%	0.31
Dyslipidemia	67,44%	31, 40.8%	36, 43.9%	0.92
History of AMI	46, 30%	26, 34.2%	20, 24.4%	0.11
History of PCI	48, 31%	27, 35.5%	21, 25.6%	0.11
History of CABG	7, 4.5%	4, 5.3%	3, 3.7%	0.57
History of stroke	4, 2.6%	2, 2.6%	2, 2.4%	0.90
**BASELINE CLINICAL STATUS**				
Heart rate [beats/min]	75 (66-88)	70 (65-75)	75 (70-80)	0.04
Systolic BP	140 (123-160)	140 (120-160)	140 (125-160)	0.95
EF [%]	55 (45-60)	55 (50-60)	55 (40-60)	0.16
NYHA class III or IV	4, 2.6%	0, 0%	4, 4.9%	0.10
Killip class				0.60
1	139, 90%	66, 86.8%	73, 89.0%	
2	14, 9.1%	5, 6.6%	9, 11.0%	
3	1, 0.6%	1, 1.3%	0, 0%	
4	0, 0%	0, 0%	0, 0%	
GRACE	124 (104-146)	116 (100-136)	132 (110-158)	0.003
**LABORATORY PARAMETERS**				

Hs-TnT T0 [ng/l]	33 (13-143)	21 (10-114)	57 (19-179)	0.006
Hs-TnT T6 [ng/l]	75 (16-397)	27 (13-129)	175 (29-901)	<0.001
Hs-TnT max [ng/l]	105 (23-530)	43 (17-209)	204 (31-1115)	0.001
CK-MB T0 [IU/l]	20 (15-30)	18 (14-27)	22 (17-35)	0.008
CK-MB T6 [IU/l]	21 (14-45)	17 (13-27)	30 (16-72)	<0.001
CK-MB max [IU/l]	27 (18-53)	24 (16-33)	33 (22-86)	<0.001
NT-proBNP [pg/ml]	350 (163-1074)	248 (122-640)	541 (180-1468)	0.003
CRP [mg/l]	2.9 (1.3-5.5)	1.9 (0.8-4)	3.8 (1.7-6.5)	0.003
Leukocytosis [10^3/µl]	8.4 (6.9-10.3)	7.6 (6.2-9.2)	8.9 (7.5-11)	<0.001
Hemoglobin [g/dl]	14 (13-15)	14 (13-15)	14 (13-15)	0.76
GFR [ml/min/1.73m^2]	92 (76-110)	102 (78-117)	90 (67-99)	0.008
**IN-HOSPITAL PARAMETERS**				
Diagnosis of CAD	116, 75%	52, 68.4%	64, 78%	0.49
Medical therapy	38, 25%	21, 27.6%	17, 20.7%	0.23
PCI	90, 58%	34, 44.7%	56, 68.3%	0.008
CABG	33, 21%	18, 23.7%	15, 18.3%	0.31
Catecholamines	4, 2.6%	0, 0%	4, 4.9%	0.06
ASA	141, 92%	64, 84.2%	77, 93.9%	0.24
DAPT	99, 64%	41, 53.9%	58, 70.7%	0.08
B-blocker	134, 87%	66, 86.8%	68, 82.9%	0.09
ACE inhibitor	126, 82%	61, 80.3%	65, 79.3%	0.35
Statin	135, 88%	64, 84.2%	72, 87.8%	0.80
Diuretic	45, 29%	22, 28.9%	23, 28.0%	0.72
Ca-blocker	39, 25%	18, 23.7%	21, 25.6%	0.94
Nitroglycerin	17, 11%	8, 10.5%	9, 11.0%	0.98
**FINAL DIAGNOSIS**				
Unstable Angina	30, 20%	18, 23.7%	12, 14.6%	0.11
NSTEMI	105, 68%	41, 53.9%	64, 78.0%	0.005

Data presented as n, % or median (25th-75th interquartile range).

ACE – angiotensin converting enzyme, AMI – acute myocardial infarction, ASA – acetylsalicylic acid, BMI – body mass index, BP – blood pressure, CABG – coronary artery bypass grafting, CAD – coronary artery disease, CK-MB – creatine kinase myocardial bound, CRP – C-reactive protein, DAPT – dual antiplatelet treatment, EF – ejection fraction, GFR – glomerular filtration ratio, GRACE – Global Registry for Acute Coronary Events, Hs-TnT – high sensitive troponin T, NSTEMI – non-ST-segment elevation myocardial infarction, NT-proBNP – N-terminal pro-B-type natriuretic peptide, NYHA – New York Heart Association, PAD – peripheral artery disease, PCI – percutaneous coronary intervention.

Figure S1. Survival curves or copeptin with the cutoff 10 pmol/l.

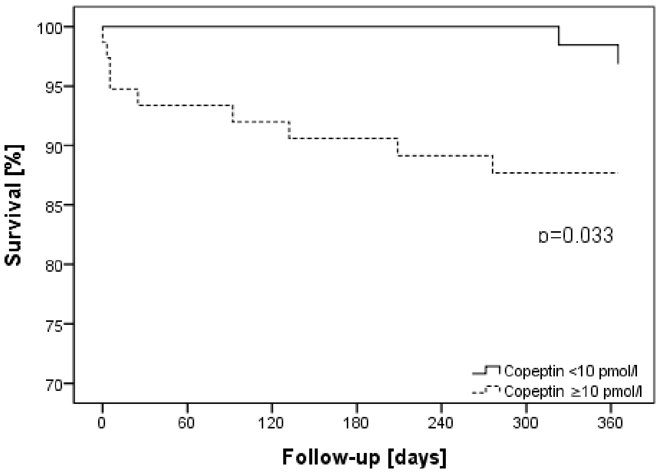

Survival rate for copeptin negative vs. copeptin positive patients at six months: 71/71 vs. 73/80 patients, p=0.01, and at one year: 69/71 vs. 67/76 patients, p=0.033.

Supplementary statistical analysis – the taxonomic analysis.

In order to analyse prognostic accuracy of copeptin in a multivariate manner and describe classificatory similarity of patients based on selected parameters, the taxonomic analysis (focus analysis) was used. The method was extensively described previously [1]. In brief, the focus defines a group of patients with similar parameters, identified with a similarity function. The taxonomic distance within the function was described with the use of Marczewski-Steinhaus method [2]. Calculated taxonomic distance define so called dendrograms - tree diagrams showing distance and relationship between parameters characteristic for studied patients based on assumed criteria. The taxonomic distance is reflected in the height of lines (y axis). Relations and distances allow to identify “localization” of patients in the space of analysed parameters, from minimal to maximal variation between those parameters (x axis; each point at the end of bottom lines represents one patient). The value of taxonomic distance defines the focus, which is reflected in the similarity in analysed parameters. The focus allows to assess differences in the values of parameters characteristic for patients, which are maximally homogenic at given level of distance within the focus and maximally

heterogenic inbetween. Taxonomic analysis included parameters which reached the p value of 0.01 or less in the Cox regression model from the main analysis.

Results of taxonomic analysis

The taxonomic analysis for a data set combined of copeptin, age and GFR was performed. A dendrogram shows the differentiation of patients in two types regarding pre-specified data (**Figure S2**). Patients characterized with higher median copeptin levels, older age and lower GFR (Type 1, n=55) than other patients (Type 2, n=99) died significantly more frequently than type 2 patients. There was no difference in the occurrence of MACCE at six months or one year between both types of patients. The combination of higher copeptin/older age/lower GFR identified a group with higher admission and follow-up NT-proBNP levels, lower baseline ejection fraction and higher risk according to the GRACE risk score (**Table S2**).

A set of baseline and simple characteristics – copeptin, age and GFR – carry high potential to stratify patients as high- and low-risk. Accordingly, young patients (median 60 years old) with low copeptin levels (median 9.7 pmol/l) and good renal function (median GRF 106 ml/kg/1.73m^2) had a very low rates of mortality (1% and 3% at six months and one year, respectively). On the other hand, approximately one in every six older patients with impaired renal function and high copeptin levels died with the within one year.

Table S2. Outcomes of the taxonomic analysis.

Characteristic	Type 1, n=55	Type 2, n=99	p value
**Copeptin** [pmol/l]	15 (6.7;64)	9.7 (4;17)	0.005
**Age** [years]	72 (63;80)	60 (54;67)	<0.001
**GFR** [ml/kg/1.73m^2]	68 ± 18	106 ± 20	<0.001
Baseline data			
EF [%]	50 (40;55)	55 (50;60)	0.001
Hs-TnT [ng/l]	41 (19;162)	32 (9.7;143)	0.06
NT-proBNP [pg/ml]	627 (267;1610)	248 (116;756)	<0.001
GRACE risk score	146 (129;170)	110 (96;130)	<0.001
ACS	92% (51/55)	85% (84/99)	0.20
Six months follow-up			
Mortality	12% (7/55)	1% (1/99)	0.003
MACCE	15% (8/55)	11% (11/99)	0.61
One year follow-up			
Mortality	15% (8/55)	3% (3/99)	0.02
MACCE	22% (12/55)	14% (14/99)	0.26
NT-proBNP [pg/ml]	351 (141;540)	146 (77;293)	0.003
EF [%]	50 (47;55)	55 (49;58)	0.19

Data are presented as median (25th-75th interquartile range), n ± SD or % (n).

ACS – acute coronary syndrome, EF – ejection fraction, GFR – glomerular filtration ratio, GRACE – Global Registry of Acute Coronary Events, hs-TnT – high-sensitive troponin T, MACCE – major adverse cardiac and cerebrovascular events, NT-proBNP – N-terminal pro-B-type natriuretic peptide, SD – standard deviation.

Figure S2. Dendrogram – taxonomic analysis.

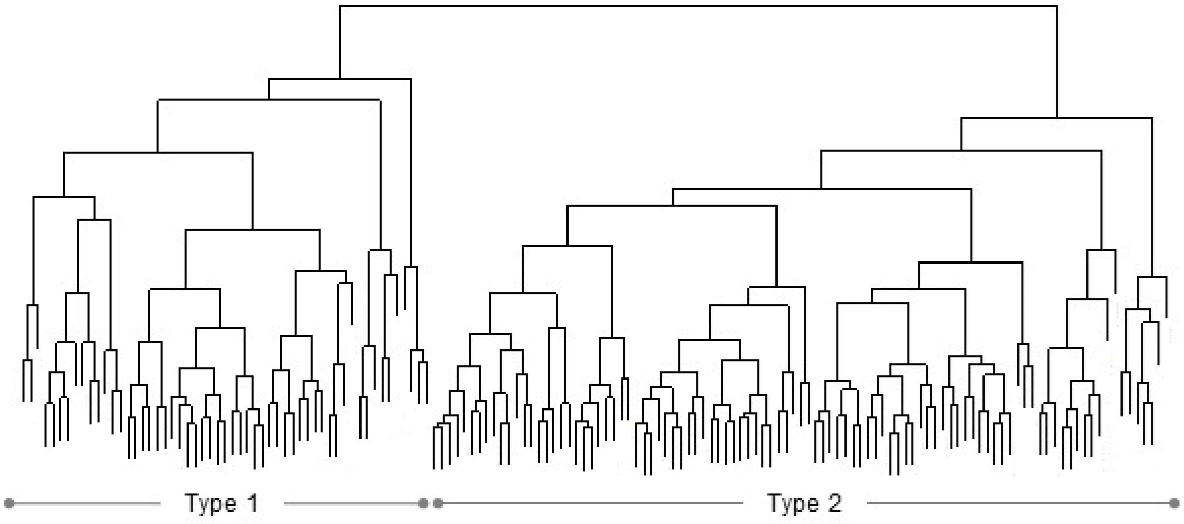

Each line at the bottom level represents one patient. Along with clustering of patients by the taxonomic algorithm, patients form groups with maximal similarity in predefined variables: copeptin, age, GFR (next levels of the diagram). Finally two main groups (types) of patients were identified (upper level of the diagram), with maximal heterogeneity in predefined variables.

References

1. Morawiec B, Kawecki D, Ho L, Chun Tat L, Muller O, Nowalany-Kozielska E (2016) COPeptin for diagnosis and prediction in Acute Coronary Syndrome (COPACS) Study: design and objectives. Adv Interv Cardiol 12 (46):1-4
2. Marczewski. E, Steinhaus. H (1958) On a certain distance of sets and the corresponding distance of functions. Colloquium Mathematicum (6):319-327
